# Case Report: Diagnostic pitfalls in soft tissue tumors: synovial sarcoma masquerading as venous malformation

**DOI:** 10.3389/fonc.2025.1615945

**Published:** 2025-08-25

**Authors:** Xuan Jiang, Li Hu, Hui Chen, Xi Yang, Xiaoxi Lin

**Affiliations:** ^1^ Department of Plastic and Reconstructive Surgery, Shanghai Ninth People’s Hospital, Shanghai Jiao Tong University School of Medicine, Shanghai, China; ^2^ Department of Laser and Aesthetic Medicine, Shanghai Ninth People’s Hospital, Shanghai Jiao Tong University School of Medicine, Shanghai, China

**Keywords:** synovial sarcoma, intramuscular venous malformation, MRI, pathological examination, SS18

## Abstract

**Introduction:**

Synovial sarcoma (SS) is one of the most prevalent malignant soft tissue sarcomas in children and adolescents. Pediatric populations often present with atypical features, complicating the differentiation from benign intramuscular venous malformations (VMs).

**​​Case presentation:**

An 11-year-old male with a four-year history of progressive right plantar pain and a compressible intramuscular mass. The initial magnetic resonance imaging (MRI) findings suggest VM, due to high signal in T2-weighted images. Sclerotherapy under digital subtraction angiography (DSA) revealed unexpected hyper-vascularity, prompting surgical exploration. Histopathology demonstrated spindle and epithelioid cells with hemangiopericytoma-like morphology and mitotic activity, while SS18-SSX1 gene rearrangement confirmed SS. Chemotherapy was then administered, without recurrence over two years.

**​​Conclusion:**

SS may clinically and radiographically mimic benign vascular anomalies, particularly in children. Discrepancies in vascular dynamics on DSA and atypical imaging features warrant suspicion for malignancy. Early histopathological validation is critical to prevent diagnostic delays, optimize multimodal therapy, and improve outcomes in this aggressive tumor.

## Introduction

Synovial sarcoma is an aggressive malignancy accounting for 10-20% of soft tissue sarcomas in adolescents and young adults, with a median diagnostic age of 35 years. Approximately 70% of cases demonstrate a predilection for extremity involvement, particularly in the lower limbs (1). Clinically, SS manifests as a slowly enlarging, painful intramuscular mass, often misinterpreted as benign lesions due to its indolent progression. Early symptoms include localized tenderness and functional impairment, while advanced stages may present with neurovascular compression or restricted mobility (2). MRI serves as the modality of choice for SS evaluation, providing superior soft tissue contrast differentiation, multiplanar lesion characterization, and precise delineation of neurovascular bundle infiltration and lymphatic dissemination (3). Diagnostic imaging reveals T2-weighted hyperintense lesions on MRI, frequently misattributed to VMs, in which case histopathological confirmation is necessary. Definitive diagnosis relies on identifying biphasic spindle-epithelioid cell morphology and SS18-SSX fusion gene detection. The main treatment for SS is wide surgical excision with adjuvant or neoadjuvant radiotherapy, and chemotherapy, while SS is moderately sensitive to cytotoxic chemotherapy with agents such as ifosfamide and anthracyclines (4).

We report a case of an 11-year-old male with clinical symptoms and imaging features similar to VM who received related treatment.

## Case presentation

**Figure 1:**
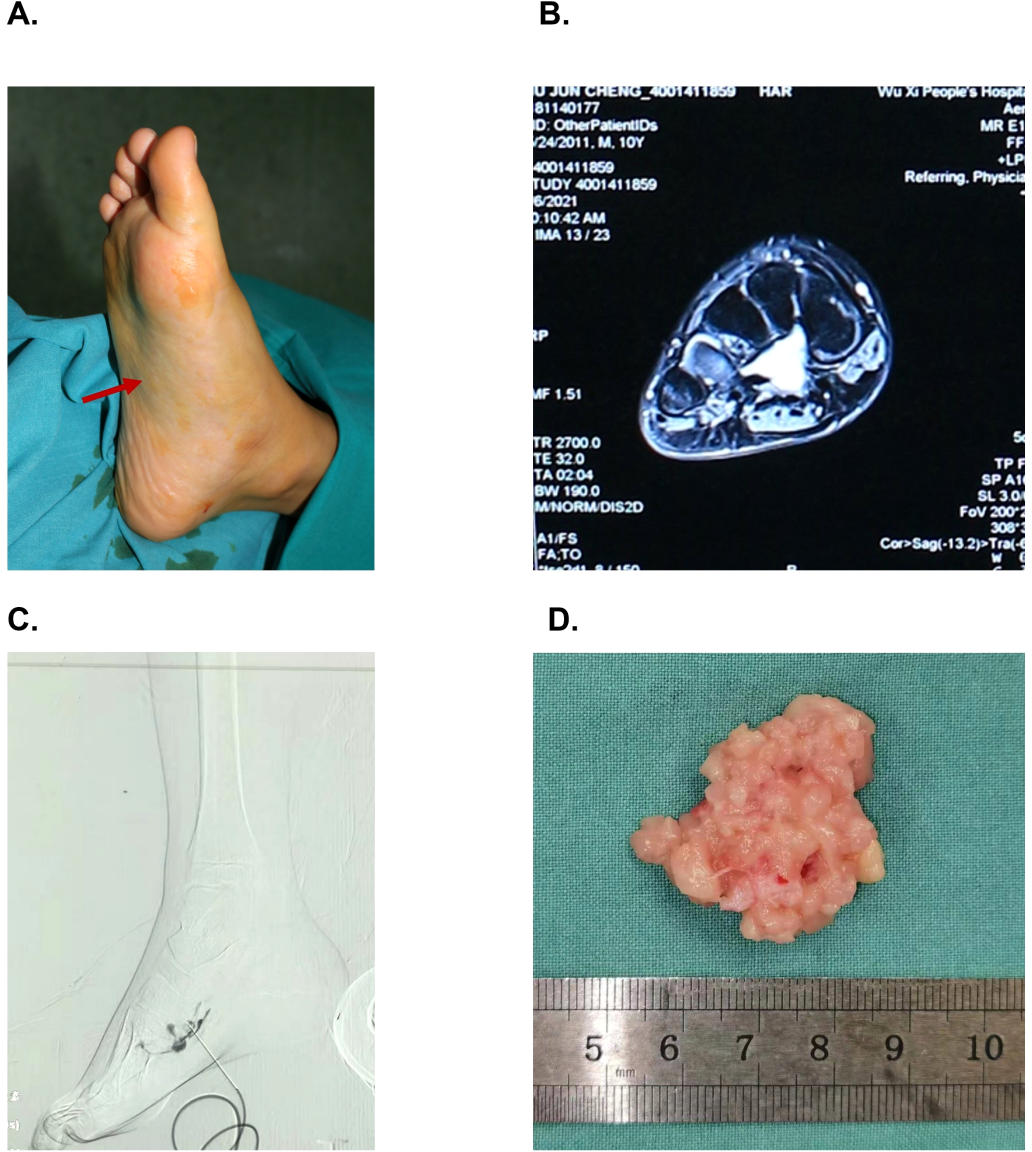
Patient diagnosis and management. The patient presented with a painful mass in right plantar **(A)**. MRI revealed a high signal on T2-weighted enhanced sequence **(B)**. The initial diagnosis was venous malformation, and sclerotherapy under DSA was performed, followed by surgical excision **(C)**. Pathological biopsy of the lesion was subsequently established **(D)**.

An 11-year-old male had a four-year history of right plantar pain worsening progressively, and an intramuscular mass enlarging gradually which had no obvious inducement. Limping occurred about 1 year ago. He has gained weight as child of the same age since the onset. On physical examination, a soft and compressible mass was found in the right plantar region, with no obvious boundary, pulsation, or paresthesia. A high-signal intensity mass on T2-weighted fat-suppression images was revealed through MRI, suggesting vascular origin (**Figure 1**). He was diagnosed as intramuscular VM.

Considering the preliminary diagnosis, the patient underwent sclerotherapy under DSA. However, the lesion showed hyper-vascularity during the procedure, which was inconsistent with the slow-flow characteristics of VMs, raising suspicion for a different pathology possibility.

**Figure 2:**
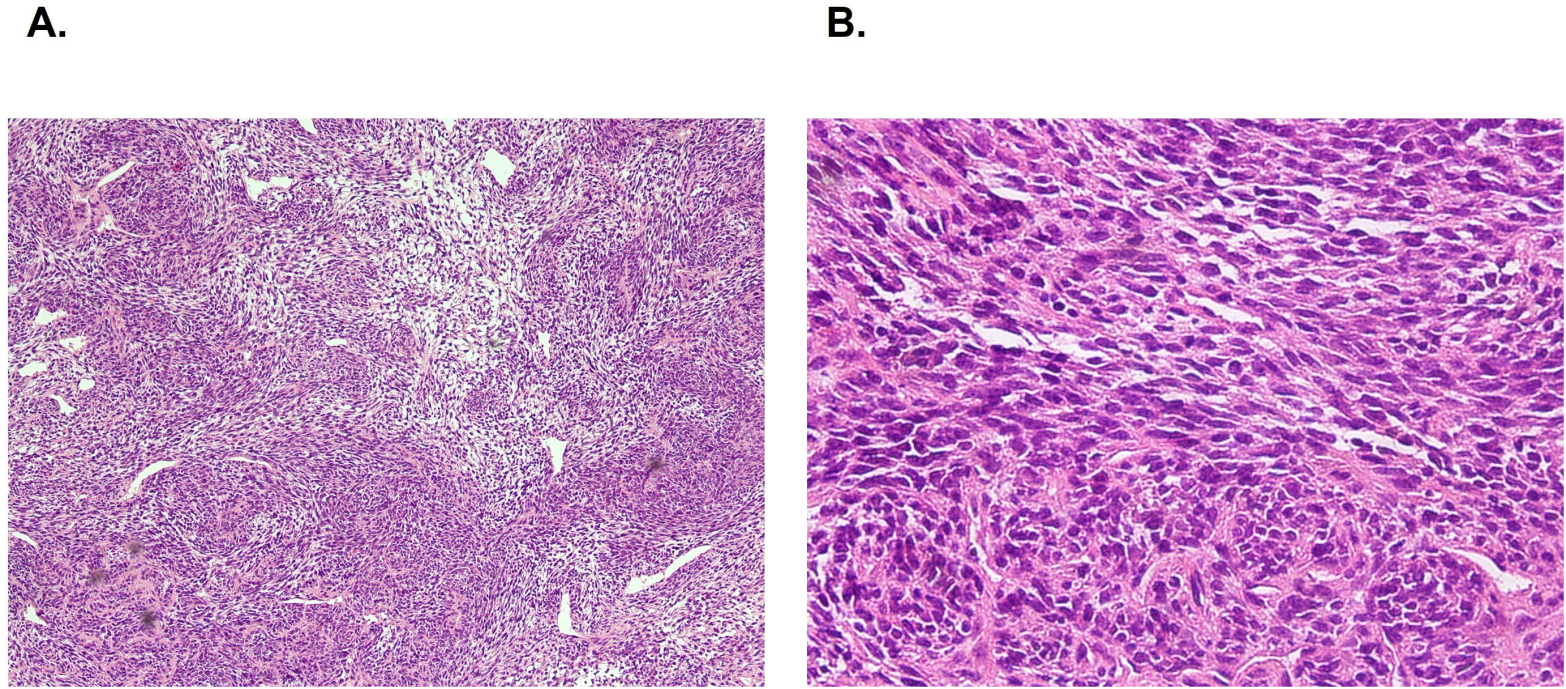
Histology of spindle cell synovial sarcoma of right plantar. The tumor is mesenchymal malignancy, characterized by a mix of spindle-shaped and epithelioid cells. Hemangiopericytoma like image and mitotic figures can be observed. The figure demonstrates H&E-stained sections at magnifications of 100× **(A)** and 200× **(B)**.

Consequently, surgical exploration was conducted to obtain a definitive diagnosis. A yellowish white lipoid frail soft tissue was found below flexor digitorum brevis and abductor hallucis. Both the surrounding muscles and fascia spaces were involved and no obvious boundary was observed (**Figure 1**). Pathological sections showed the cell characteristics of the mesenchymal malignant tumor tissue, including spindle cells and epithelial cells. Hemangiopericytoma like image and mitotic figures could be seen (**Figure 2**). Immunohistochemical analysis demonstrated positivity for SS-characteristic markers, including diffuse positivity for TLE1 and focal positivity for both CK and CK7. The discovery of fluorescence *in situ* hybridization (FISH) showed the SS18 (18q11) gene rearrangement, confirming the diagnosis of SS.

After confirming the diagnosis, extended resection was performed. Moreover, chemotherapy was initiated with a VAC regimen (vincristine, actinomycin D, and cyclophosphamide), administered six cycles over six months. The patient tolerated the treatment well, with relief of pain and no notable adverse effects. No recurrence was observed during the two years of follow-up.

## Discussion

Distinguishing intramuscular SS from VM is a significant diagnostic challenge, especially in pediatric patients who often present atypically. Intramuscular VMs are benign vascular anomalies characterized by dilated, endothelium-lined venous channels embedded within muscle tissue. Typically, these lesions are soft, compressible masses (5). MRI usually show hyperintensity on T2-weighted sequence (6). SS is a neoplasm originating from mesenchymal tissue, often show similar clinical and imaging features that can be confused with benign conditions like VMs. SS typically present as soft, often tender masses; however, unlike venous malformations, they are generally non-compressible. In SS, a heterogeneous appearance on MRI frequently appears, with high T2 signal due to cystic changes. VM exhibit progressive heterogeneous enhancement, whereas SS typically demonstrates homogeneous enhancement. Moreover, unlike VMs, significant enhancement on contrast imaging and hyper-vascular response on DSA is shown in SS. These are important characteristics for raising suspicion in the case and called for further investigation, as the initial diagnosis based on only imaging was misleading (7).

Histopathological analysis remains the gold standard for SS diagnosis. The SS18 (18q11) gene rearrangement is a hallmark of SS and provides a definitive diagnostic criterion (8). Since SS is a highly aggressive tumor, it’s necessary to make early accurate diagnoses so that appropriate treatments can be arranged as quickly as possible. Delays in diagnosis, as seen in cases initially misdiagnosed as benign entities, can result in disease progression and worse prognose (9).

The optimal management of extremity soft tissue sarcomas needs establishing a definitive pathological diagnosis preoperatively whenever possible, supported by detailed imaging to delineate the tumor’s relationship with surrounding tissues prior to formulating a surgical strategy (10, 11). Based on oncological surgical principles, specifically achieving a R0 resection, remains the cornerstone of treatment, as wide excision significantly reduces both local recurrence rates and mortality (10, 11). If R0 resection cannot be attained, neoadjuvant chemotherapy and/or radiotherapy is warranted. Doxorubicin (ADM) and Ifosfamide (IFO) represent the foundational chemotherapeutic agents for soft tissue sarcomas, while pediatric patients, demonstrating greater chemosensitivity, often derive significant benefit from the VAC regimen (11, 12). Prognosis is determined by factors such as post-treatment recurrence, metastasis occurrence, and time to disease progression, with the initial tumor stage, histological grade, and adequacy of initial therapy being primary determinants influencing recurrence and metastatic risk (11).

## Conclusion

This case is remarkable as it emphasizes the potential defects of relying only on non-invasive imaging for soft tissue masses, particularly in pediatric patients whose malignancies are less commonly suspected. For patients with MRI features mimicking VMs, biopsy should be performed to obtain a definitive histopathological diagnosis to differentiate from soft tissue tumors.

## Data Availability

The original contributions presented in the study are included in the article/supplementary material. Further inquiries can be directed to the corresponding authors.
